# Functional group and diversity analysis of BIOFACQUIM: A Mexican natural product database

**DOI:** 10.12688/f1000research.21540.2

**Published:** 2020-06-08

**Authors:** Norberto Sánchez-Cruz, B. Angélica Pilón-Jiménez, José L. Medina-Franco

**Affiliations:** 1 Department of Pharmacy, School of Chemistry, National Autonomous University of Mexico, Mexico City, Mexico City, 04510, Mexico

**Keywords:** Consensus Diversity Plot, compound databases, data mining, diversity, natural products, functional groups, in silico

## Abstract

**Background:** Natural product databases are important in drug discovery and other research areas. An analysis of its structural content, as well as functional group occurrence, provides a useful overview, as well as a means of comparison with related databases. BIOFACQUIM is an emerging database of natural products characterized and isolated in Mexico. Herein, we discuss the results of a first systematic functional group analysis and global diversity of an updated version of BIOFACQUIM.

**Methods:** BIOFACQUIM was augmented through a literature search and data curation. A structural content analysis of the dataset was performed. This involved a functional group analysis with a novel algorithm to automatically identify all functional groups in a molecule and an assessment of the global diversity using consensus diversity plots. To this end, BIOFACQUIM was compared to two major and large databases: ChEMBL 25, and a herein assembled collection of natural products with 169,839 unique compounds.

**Results:** The structural content analysis showed that 15.7% of compounds and 11.6% of scaffolds present in the current version of BIOFACQUIM have not been reported in the other large reference datasets. It also gave a diversity increase in terms of scaffolds and molecular fingerprints regarding the previous version of the dataset, as well as a higher similarity to the assembled collection of natural products than to ChEMBL 25, in terms of diversity and frequent functional groups.

**Conclusions:** A total of 148 natural products were added to BIOFACQUIM, which meant a diversity increase in terms of scaffolds and fingerprints. Regardless of its relatively small size, there are a significant number of compounds and scaffolds that are not present in the reference datasets, showing that curated databases of natural products, such as BIOFACQUIM, can serve as a starting point to increase the biologically relevant chemical space.

## Introduction

Natural product-based drug discovery continues to be an important part of drug discovery. Recently, the synergy between natural product research with molecular modeling and chemoinformatics is gaining importance, speeding up the drug discovery process ^1, 2^. As part of these synergistic efforts, curated databases of natural products have an important role as they are major tools for data mining, hypothesis generation, and starting points of virtual screening. There are several databases of natural products in the public domain as reviewed recently ^3^. Our research group has reported initial efforts to assemble a database of natural products from Mexico called BIOFACQUIM ^4^. As part of that work, scaffold content and chemical space diversity were examined. However, detailed functional group (FG) content analysis, which has been proven to be valuable to characterize compound databases ^5^, in particular from natural sources ^6^, has not been reported for BIOFACQUIM. One of the main reasons is that most of the currently available software employed for identification of functional groups rely on a predefined set of substructures, even when it has been established that one of the major features that discriminate natural products from synthetic compounds are their unique functional groups.

Herein, we report a functional group content analysis of an updated version of BIOFACQUIM. We employed a validated and novel algorithm that identifies all functional groups in a molecule. As part of the analysis and to compare the results of BIOFACQUIM we also discuss the functional group contents of other large and related databases in the public domain, namely ChEMBL 25 ^7^ and a herein assembled collection of natural products (NPs) with 169,839 compounds.

## Methods

### Databases and data curation

As described elsewhere, the first version of BIOFACQUIM was developed as a proof-of-concept database applying several filters to include compounds ^4^. Briefly, the database was focused on natural products published between 2000 and 2018 by research groups in a major Mexican institution in eight indexed journals: *Journal of Ethnopharmacology*, *Natural Products Research*, *Journal of Agricultural and Food Chemistry*, *Journal of Natural Products*, *Planta Medica*, *Phytochemistry, Natural Product Letters*, and *Molecules*. As additional criteria for inclusion of compounds and to increase the quality and reliability of the contents of the database, the procedure for the isolation, purification, and characterization of the natural product should have been described in the article. In this work, we expanded the contents of the BIOFACQUIM database to further explore the diversity of natural products from Mexico.

The second version of BIOFACQUIM was assembled using the same methodology described to develop the first version ^4^ extending the date of publication to 2019. To achieve the objective of being representative of Mexico, one additional criterion was considered, including only compounds collected in Mexico at any of its institutions (universities, research laboratories and research centers). For the new version of the database, the same procedure for the curation was performed ^4^, using Molecular Operating Environment (MOE) software, although this procedure can be performed using open source software, such as MolVS and RDKit. The updated and curated version of BIOFACQUIM contains 531 compounds.

Table 1 summarizes the information of BIOFACQUIM and other major compound databases used in this work as reference: ChEMBL 25 as a representative example of the biologically tested chemical space with 1,667,509 unique compounds; and a collection of known natural products with a total of 168,030 molecules. The reference natural product collection was assembled from three general and publicly available natural products databases: the Universal Natural Products Database (UNPD) ^8^, the Natural Products Atlas ^9^ and Natural Products in PubChem Substance Database ^10^. The data sets were curated using the same procedure. Briefly, compounds were standardized and those consisting of multiple components were split and the largest component was retained. Compounds consisting of any element other than H, B, C, N, O, F, Si, P, S, Cl, Se, Br and I, as well as compounds with valence errors, were removed from the data set. The remaining compounds were neutralized and reionized to subsequently generate a canonical tautomer. Finally, canonical simplified molecular-input line-entry system (SMILES) (ignoring stereochemistry information) were generated as molecular representation and duplicate structures in the context of each database were removed. The entire process was performed by using the functions Standardizer, LargestFragmentChoser, Uncharger, Reionizer and TautomerCanonicalizer implemented in the molecule validation and standardization tool MolVS for the open source cheminformatics toolkit RDKit. The code is available at GitHub ( https://github.com/DIFACQUIM/IFG_General).

**Table 1.** Compound databases analyzed in this work and summary statistics of their diversity.

Database	Size (compounds)	Median similarity (MACCS keys - 166 bits)	Median similarity (Morgan2 - 1024 bits)	Mean distance (PCP)	Scaffold diversity (AUC)	Scaffold diversity (F _50_)
BIOFACQUIM V1	403	0.457	0.123	3.648	0.725	0.165
BIOFACQUIM V2	503	0.446	0.119	3.319	0.710	0.171
Natural products	168,030	0.422	0.111	3.775	0.830	0.032
ChEMBL 25	1,667,509	0.382	0.117	2.187	0.809	0.057

PCP: physicochemical properties; AUC: area under the cyclic system retrieval curve.

### Databases overlap

Overlap of BIOFACQUIM with the databases selected as reference was assessed in terms of three different structural levels: compounds, scaffolds, and functional groups. Compound overlap was determined in terms of canonical SMILES. For scaffold comparison we use the definition proposed by Bemis and Murcko ^11^ as implemented in RDKit, while for functional group overlap we selected the recently published definition and implementation suggested by Ertl ^5^. For each structural level, we identified the unique structures belonging to each dataset as well as those belonging to two or three of them.

### Functional group analysis

For the functional group content analysis we selected the algorithm recently described by Ertl ^5^, which is able to identify all functional groups in a molecule based on an iterative marching through its atoms. In short, the proposed algorithm identifies all heteroatoms in a molecule, all atoms connected by multiple bonds as well as the atoms in oxirane, aziridine, and thiirane rings. Afterwards, all connected atoms are joined together to form a functional group. Single aromatic heteroatoms are retained only if they are connected to an additional aliphatic functionality. Finally, a generalization scheme is applied in which for a defined list of common FGs, information about the parent carbon is retained (e.g. to differentiate between alcohols and phenols) as well as hydrogen atoms (e.g. to differentiate between aldehydes and ketones). The method is fully described in 5. An open source version of this algorithm is available for Python ( https://github.com/rdkit/rdkit/tree/master/Contrib/IFG); however, it does not cover the generalization scheme proposed originally. To this end and based on the code available, we implemented with RDKit a fragmentation approach considering keeping the parent carbon and hydrogen atoms proposed originally, were the remaining carbon atoms are replaced by dummy atoms. This implementation works over a SMILES string and returns a list with the canonical SMILES of the FGs identified in the molecule. The code is freely available at GitHub ( https://github.com/DIFACQUIM/IFG_General). After determining the FGs content of the different datasets, we compare the proportion of the most frequent FGs at each library.

### Complexity analysis

The fraction of carbon atoms that are sp ^3^ hybridized (F-sp3) is a common metric to quantify molecular complexity ^12^. Higher values for this descriptor have been associated to an improved binding selectivity of compounds ^13^. In order to compare the complexity of BIOFACQUIM with the data sets selected as reference, we computed the F-sp3, as implemented in RDKit, for all compounds in the three data sets and compared its distribution among libraries.

### Chemical space visualization

In order to generate a visual representation of the chemical space covered by the analyzed databases, we selected a recently proposed method, named TMAP ^14^ (Tree Manifold Approximation and Projection). This method enables the visualization of up to millions of data points with high dimensionality as a two-dimensional tree and has shown to be better suited than t-distributed Stochastic Neighbor Embedding ^15^ (t-SNE) and Uniform Manifold Approximation and Projection ^16^ (UMAP) for the exploration of large datasets. TMAP consists in four phases: (I) the input data are indexed in an local sensitive hashing forest data structure, using *l* prefix trees and *d* hash functions in encoding the data, (II) an undirected weighted *c*-approximate *k*-nearest neighbor graph ( *c*- *k*-NNG) is constructed from the indexed data points with the Jaccard distances between vertices used as edges weights, (III) a minimum spanning tree (MST) is constructed for the weighted *c*- *k*-NNG using Kruskal’s algorithm and (IV) a layout for the resulting MST is constructed by using a spring-electrical model layout algorithm with multilevel multipole-based force approximation as provided by the modular C++ library, open graph drawing framework ^17^ (OGDF).

For the description of compounds, we selected Morgan fingerprints with radius 2 (Morgan2, 1024-bits) as implemented in RDKit. For the generation of the TMAP, the input data was encoded by 1024 hash functions and indexed using 64 prefix trees. The weighted *c*- *k*-NNG was built using the 5 nearest neighbors and a factor of 20 for the LSH forest query algorithm. For the layout generation a node size of 0.01 was selected while all the remaining parameters were set to default. These calculations were done using the TMAP python package and all the charts were generated using the matplotlib library ^18^.

### Global diversity

The “global” or total diversity of the datasets was analyzed through the Consensus Diversity (CD) Plot ^19^. A CD Plot is a two-dimensional representation of compound datasets based on four different and complementary diversity criteria: molecular fingerprints, molecular scaffolds, physicochemical properties (PCP), and size. Fingerprint-based diversity of each dataset is represented in the X-axis, while scaffold-based diversity is represented in the Y-axis, PCP-based diversity is represented as the filling of the data points using a continuous color scale and the size of the data set is represented with the size of the data points.

For this work, scaffold diversity was assessed as the area under the cyclic system retrieval curve and the fraction of chemotypes that covers 50% of the dataset (F _50_). The median of the lower triangle from the pairwise similarity matrix computed as the Tanimoto coefficient of both MACCS keys (166-bits) fingerprint and Morgan2 (1024-bits), were used as molecular fingerprint-based diversity. For PCP-based diversity, six molecular properties of pharmaceutical interest were computed for each unique compound, being averaged molecular weight (AMW) partition coefficient octanol/water (SlogP), number of hydrogen bond donors (HBD), number of hydrogen bond acceptors (HBA), number of rotatable bonds (RB), and topological polar surface area (TPSA); PCP-based diversity was measured as the mean distance of the lower triangle of the pairwise distance matrix computed as the Euclidean distance of those PCPs scaled (mean 0 and unit variance). The number of compounds in each dataset was selected as measure of the size-based diversity. PCPs were calculated as implemented in RDKit and the CD Plot was constructed using R.

## Results and discussion

### Update of BIOFACQUIM

As described in the Methods section, the updated BIOFACQUIM database contains the chemical structure of 531 compounds, all collected from Mexico. As with the first version ^4^, each molecule is annotated with information of the chemical structure, the original source of the information (Digital Object Identifier, DOI, to reference paper), kingdom, genus, and species of the organism from which the natural product was isolated, place of collection (city and state), and activity value of the reported biological activity. From the original dataset containing 423 compounds, 40 were discarded since they were not collected in Mexico, which means an increase of 148 unique compounds compared to the previous release of the database. The sources of the 531 compounds are distributed as follows: 406 from plants, 97 from fungus, 15 from propolis and 13 from marine animals.

### Database overlap

**Figure 1.**
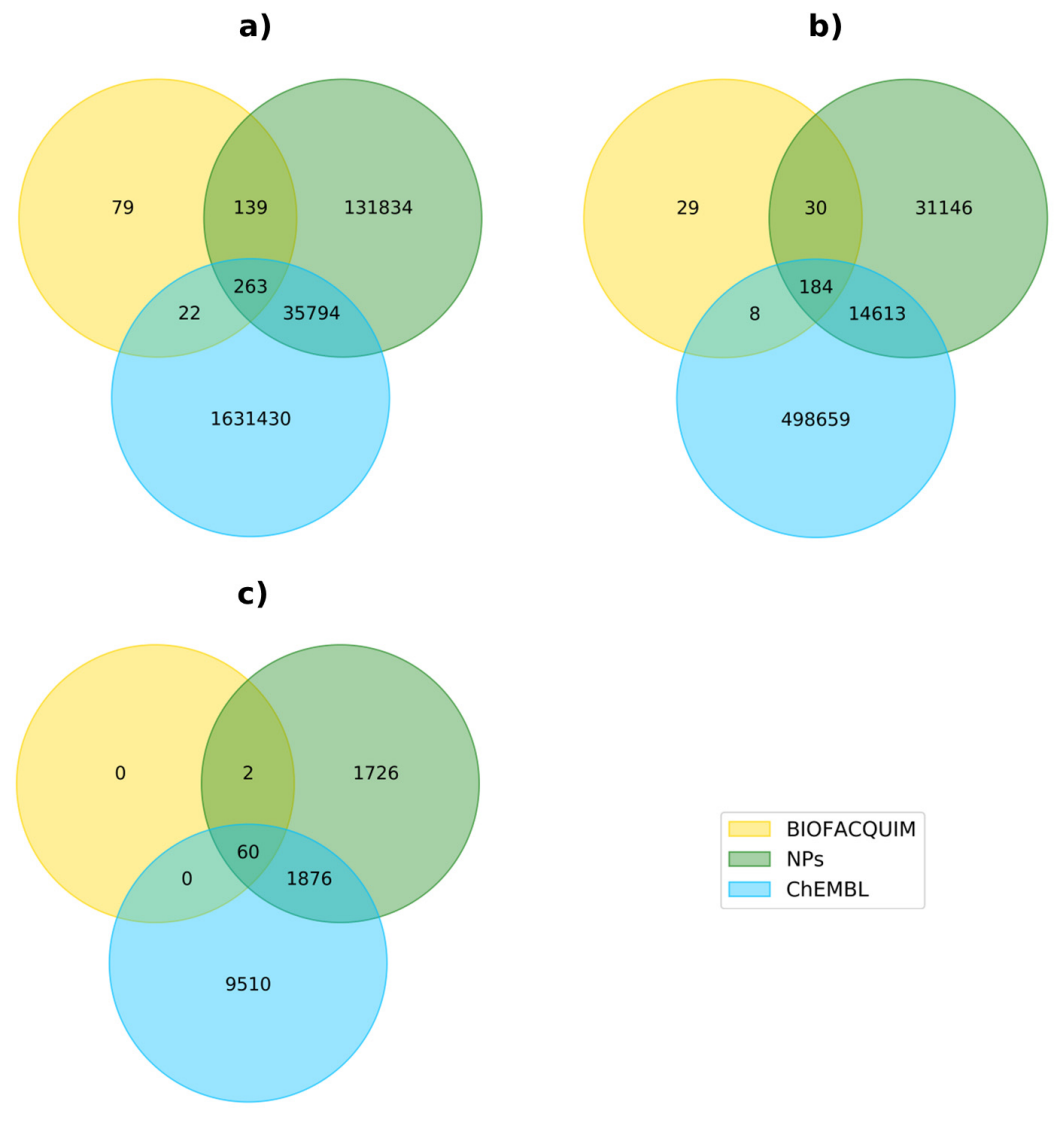
Overlap between BIOFACQUIM, reference natural products (NPs) and ChEMBL. Datasets content was analyzed in terms of ( **a**) Compounds, ( **b**) Scaffolds and ( **c**) Functional Groups.

**Figure 2.**
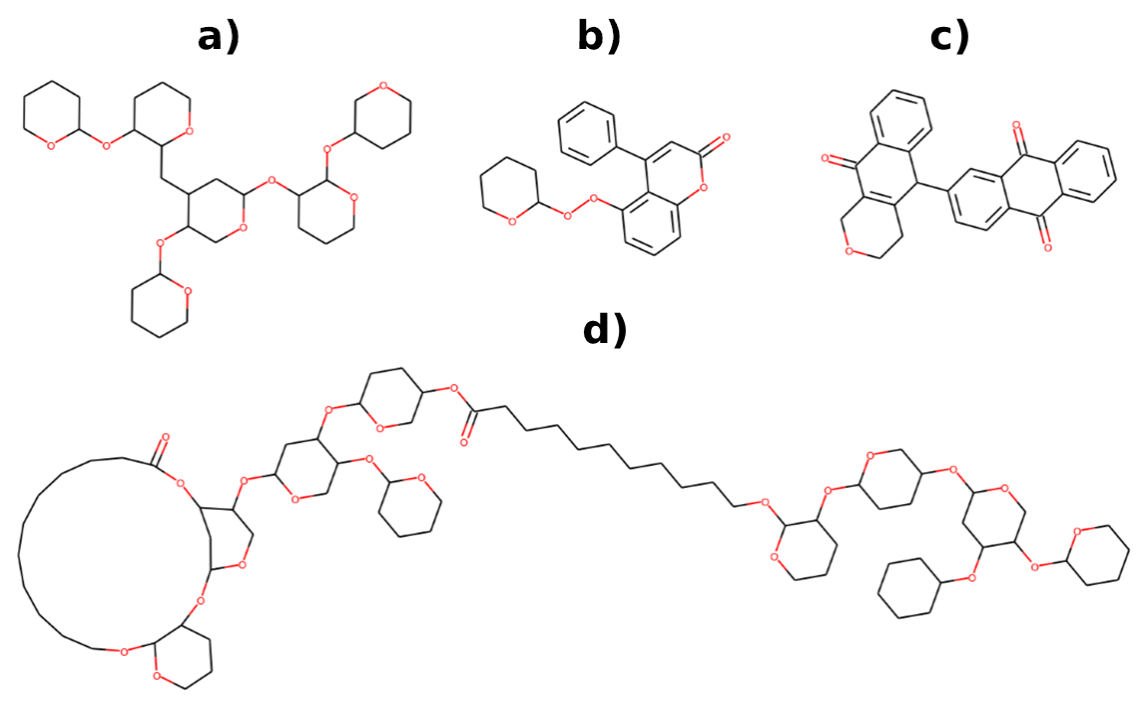
Representative unique scaffolds from BIOFACQUIM.

To assess the chemical space not covered by ChEMBL and NPs but by BIOFACQUIM, we characterized the structural content of the three datasets in terms of unique compounds, scaffolds and functional groups and determined the overlap among them. Figure 1 depicts Venn diagrams showing the overlap among those datasets. It should be noted that despite its small size in comparison with ChEMBL and NPs, 15.7% of compounds present in BIOFACQUIM have not been reported in those other major datasets, as well as 11.6% of its scaffolds. Figure 2 shows the structure of representative scaffolds identified in at least 2 compounds from BIOFACQUIM, all of them associated to compounds isolated from different plants. Scaffold **a** associated to hexasaccharides of convolvulinic and jalapinolic acids isolated from *Ipomoea purga* ^20^, scaffold **b** corresponding to glycosides of 4-phenylcoumarin isolated from *Exostema caribaeum* ^21^, scaffold **c** representing karwinaphthopyranones A2 and B3 isolated from *Karwinskia parvifolia* ^22^ and scaffold **d** associated to batatins X and XI isolated from *Ipomoea batatas* ^23^. Another remarkable observation is the fact that most of the overlap of BIOFACQUIM with the other datasets involves NPs, either alone or in combination with ChEMBL, being 79.9%, 85.3%, and 100% for compounds, scaffolds and FGs, respectively.

### Functional group analysis

A systematic analysis of functional groups was carried out over BIOFACQUIM and the two datasets selected as reference. 62, 3664, and 11446 functional groups were identified in BIOFACQUIM, NPs and ChEMBL datasets, respectively (the overlap between them is shown in Figure 1). From the total number of functional groups present in each dataset, only 12, 15, and 22 were present in at least 1% of the corresponding library (19.4%, 0.4% and 0.2%, respectively) while 30, 1879, and 5212 (48.4%, 51.3% and 45.5%, respectively) were singletons. This result is consistent with the typical power law observed in other databases ^6^. The most frequent FGs present in BIOFACQUIM are oxygen-containing FGs, being the phenolic hydroxyl group (46.1%), followed by ether (41.4%), alcohol hydroxyl group (38.4%), alkene (28.6%) and ester (26.8%), which although in a different order, are the most frequent FGs in the herein assembled NPs collection and in other natural product libraries ^6^. This is in contrast to ChEMBL in which only ether is part of the most frequent FGs while the rest of them are nitrogen containing FGs and halogens. The complete results of the FGs found of the datasets is included as *Extended data* (Supplementary File 1).

### Complexity analysis

**Figure 3.**
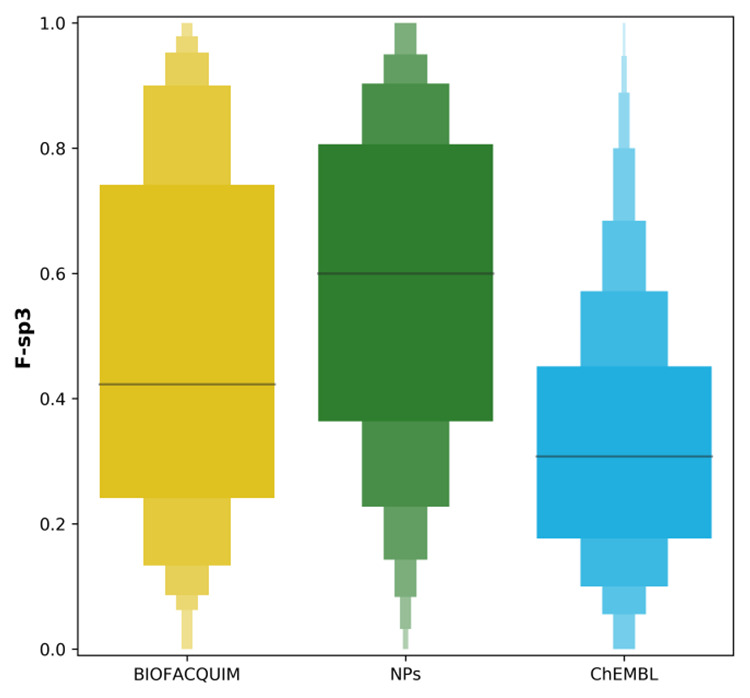
Distribution of F-sp3 for BIOFACQUIM, NPs, and ChEMBL.

As described in the Methods section, molecular complexity of BIOFACQUIM, NPs and ChEMBL data sets were assessed through the F-sp3 of its compounds. Figure 3 depicts letter plots for the distribution of this descriptor among data sets. This shows that NPs is the more complex data set overall regarding to this metric, with a median of 0.60 and a long tail towards low values. In contrast, ChEMBL is the less complex data set, with a median F-sp3 of 0.31 and a long tail towards high values. BIOFACQUIM is in an intermediate position with a median of 0.42 and a more symmetrical distribution, which is consistent with its small size in comparison to the other sets and its major overlap with NPs.

### Chemical space visualization

**Figure 4.**
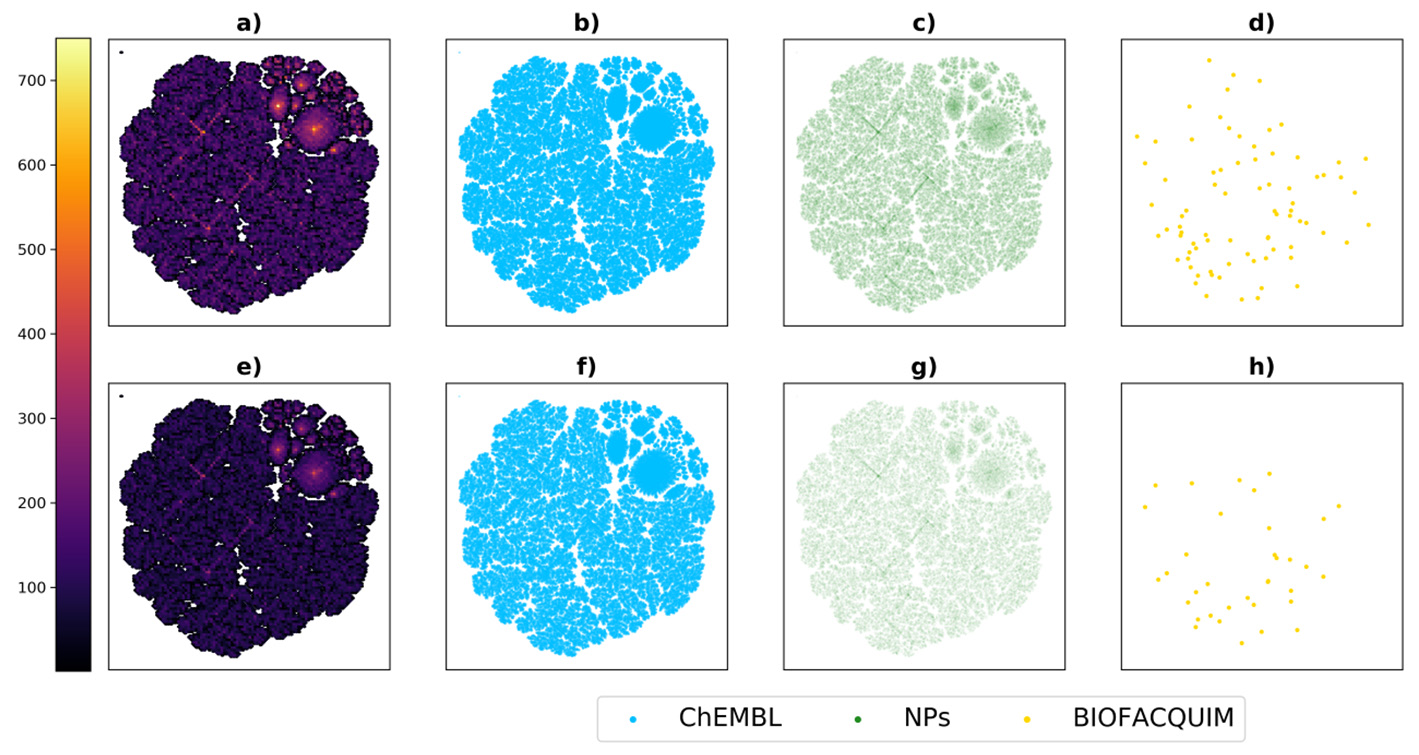
TMAP visualization of the chemical space covered by BIOFACQUIM. Comparison of BIOFACQUIM with two reference datasets. Panels **a**– **d** show all compounds in the datasets, panels **e**– **h** show drug-like compounds only. Panels **a** and **f** show the distribution of compounds from the three data sets among the chemical space in a continuous color scale.

For visualization of the chemical space of the datasets compared in this work, we built a TMAP as described in the Methods section. This method allows the representation of large datasets as a two-dimensional tree. TMAP shows the relationships among subsets of data points and data points itself as branches and sub-branches, so similar compounds and clusters tend to be close in the final representation even if the tree edges are not included, for that reason all charts in this work do not show the tree edges. Figure 4 shows a visual representation of the chemical space of the three datasets analyzed in this work, including the whole datasets and drug-like subsets. Drug-like compounds for each subset were defined those complying with the Lipinski “rule of 5” ^24^ and Veber criteria ^25^ (AMW ≤ 500, -1 ≤ SlogP ≤ 5, HBA ≤ 10, HBD ≤ 5, RB ≤ 10 and TPSA ≤ 140). For this plot, in order to better illustrate the unique compounds present in BIOFACQUIM, all compounds belonging to more than one library were assigned to a single one: ChEMBL if they belong to this dataset, NPs if they belong to this dataset but not to ChEMBL and BIOFACQUIM if they were unique for this library. Figure 4 shows that the chemical space covered by the analyzed datasets is practically defined by ChEMBL and highly focused in the upper right section of the plot, meaning that the biologically relevant space does not cover evenly the available chemical space. It is also shown that NPs cover, in a sparser manner, the same space as ChEMBL. Unique compounds from BIOFACQUIM on the other hand are distributed only in the less populated regions of the space, and even in the outer region of the plot, which implies the presence of few similar compounds in the other datasets. All these observations are equally applicable for the drug-like subsets from the original data sets, which represent 44.3%, 48.4% and 69.1% of BIOFACQUIM, NPs, and ChEMBL, respectively.

### Global diversity

**Figure 5.**
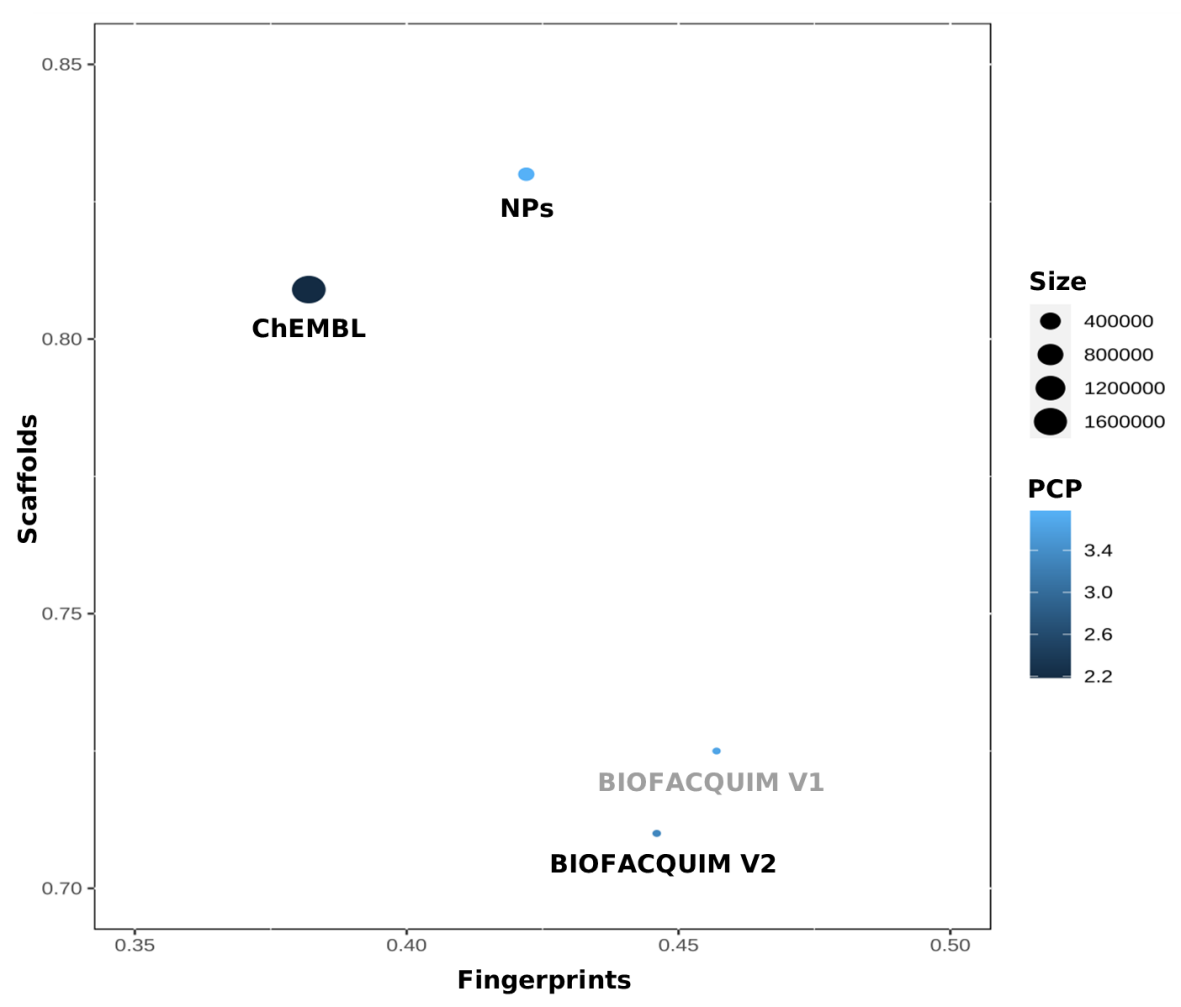
Consensus diversity plot of BIOFACQUIM.

In order to compare the chemical diversity of the current version of BIOFACQUIM with the previous one and the two datasets selected as reference, we employed a CD Plot. Figure 5 shows the plot comparing the diversity of all datasets considering four different criteria: scaffolds in the *y*-axis, molecular fingerprints in the *x*-axis, physicochemical properties as the filling of the data points in a continuous color scale, and number of compounds as the data points size. This comparison shows the relatively small size of BIOFACQUIM in comparison with the reference datasets. As compared to the previous release of BIOFACQUIM, the current version has increased its diversity in terms of scaffolds and fingerprints but decreased in terms of physicochemical properties. Also, it is shown that its diversity in terms of molecular fingerprints and physicochemical properties, although not the greatest ones of the three datasets, are closer to the ones for NPs, contrary to scaffolds, in which is the most diverse.

The molecular fingerprint diversity of each data set is represented on the *x*-axis and was defined as the median Tanimoto coefficient of MACCS keys (166-bits) fingerprint. The scaffold diversity of each database is represented on the *y*-axis and was defined as the area under the corresponding cyclic system retrieval curve. The diversity based on physicochemical properties (PCP) was defined as the mean euclidean distance of six scaled physicochemical properties (SlogP, TPSA, AMW, RB, HBD, and HBA) and is shown as the filling of the data points using a continuous color scale. The number of compounds is represented by the size of the data points.

## Conclusions

The current version of BIOFACQUIM involved the addition of 148 natural products. This was reflected in a diversity increase based on both scaffolds and molecular fingerprints. It was shown that in terms of diversity, structural content overlap and complexity, BIOFACQUIM is more similar to the assembled set of natural products than to the set of biologically tested compounds. The herein reported chemoinformatic study revealed that 44.3% of the unique compounds contained in BIOFACQUIM are focused in the drug-like space in terms of physicochemical properties. Interestingly, despite the fact of its relative small size, there were identified a significant number of compounds and scaffolds (79 and 29, respectively) that were not present in the two large sets used as reference, showing that curated databases of natural products, such as BIOFACQUIM, can serve as a starting point for the study and increase of the biologically relevant chemical space.

## Data availability

### Underlying data

Figshare: BIOFACQUIM_V2. http://doi.org/10.6084/m9.figshare.11312702

This file contains the chemical structures of 531 compounds in SDF format, alongside ID number, compound name, simplified molecular input line entry system, literature reference, kingdom, genus, species, geographical location and biological activity.

Underlying data are available under the terms of the Creative Commons Zero “No rights reserved” data waiver (CC0 1.0 Public domain dedication).

### Extended data

Figshare: Supporting information for "Functional group and diversity analysis of BIOFACQUIM: A Mexican natural product database". http://doi.org/10.6084/m9.figshare.11312735

This project contains the following extended data:

- Supplementary File 1. File with summary results of the functional group analysis.

Extended data are available under the terms of the Creative Commons Attribution 4.0 International License (CC BY 4.0).
