# Supplementary material for: Functional group and diversity analysis of BIOFACQUIM: A Mexican natural product database
Source: F1000Res. 2020 Jun 8;8:Chem Inf Sci-2071. Originally published 2019 Dec 10. [Version 2] doi: 10.12688/f1000research.21540.2 (PMC6993822; doi:10.12688/f1000research.21540.2)
Supplement: Peer review history

# Peer review: Reviewer response for version 2

**DOI:** 10.5256/f1000research.27062.r64382

**Published:** 2020-06-11

**Authors:** Johannes Kirchmair

**Affiliations:** 1 Department of Pharmaceutical Chemistry, University of Vienna, Vienna, Austria

I have no further comments or requests.

Is the work clearly and accurately presented and does it cite the current literature?

Yes

If applicable, is the statistical analysis and its interpretation appropriate?

Yes

Are all the source data underlying the results available to ensure full reproducibility?

Yes

Is the study design appropriate and is the work technically sound?

Partly

Are the conclusions drawn adequately supported by the results?

Yes

Are sufficient details of methods and analysis provided to allow replication by others?

Yes

Reviewer Expertise:

cheminformatics, natural products research

I confirm that I have read this submission and believe that I have an appropriate level of expertise to confirm that it is of an acceptable scientific standard.

# Peer review: Reviewer response for version 1

**DOI:** 10.5256/f1000research.23733.r58743

**Published:** 2020-01-30

**Authors:** Trong Tran

**Affiliations:** 1 GeneCology Research Centre, University of the Sunshine Coast, Maroochydore, Qld, Australia

The manuscript by Sánchez-Cruz *et al.* describes the cheminformatic analysis of the updated BIOFACQUIM database consisting of 531 Mexican natural products. Despite its relatively small size, the results supported the potential of using BIOFACQUIM as a good source for virtual screening in drug discovery.

 Generally, the study was well designed, and the paper was well-written. However, since stereochemistry plays a significant role in the interactions between small molecules and protein targets, I suggest the authors should add the analysis of the fraction of sp ^3^-hydridized atoms for the new BIOFACQUIM version and compare that value with those of other databases to assess its 3D unique. Can authors detail the “eight indexed journals” which was referred in the “Methods - Databases and data curation”?

 All in all, I feel this manuscript is suitable for indexing.

Is the work clearly and accurately presented and does it cite the current literature?

Yes

If applicable, is the statistical analysis and its interpretation appropriate?

Yes

Are all the source data underlying the results available to ensure full reproducibility?

Yes

Is the study design appropriate and is the work technically sound?

Yes

Are the conclusions drawn adequately supported by the results?

Yes

Are sufficient details of methods and analysis provided to allow replication by others?

Yes

Reviewer Expertise:

Natural product chemistry, Drug Discovery.

I confirm that I have read this submission and believe that I have an appropriate level of expertise to confirm that it is of an acceptable scientific standard.

# Peer review: Reviewer response for version 1

**DOI:** 10.5256/f1000research.23733.r57650

**Published:** 2020-01-24

**Authors:** W. Patrick Walters

**Affiliations:** 1 Relay Therapeutics, Cambridge, MA, USA

This paper describes an analysis of the BIOFACQUIM database of natural products derived from Mexico. The paper is well written and provides a useful overview of a resource that will be of use to medicinal and computational chemists, as well as those involved in research on natural products. The authors should be commended for making their database and associated code available as Open Source.  

 While the paper is well written, there are a few areas where changes in the text may make the presentation a bit more clear:

- The second sentence in the abstract could be changed to read: “An analysis of its structural content, as well as functional group occurrence, provides a useful overview, as well as a means of comparison with related databases."
- In the first sentence of the “Methods” section, the authors may want to consider changing “done” to “performed”.
- In the Introduction, the sentence “As part of that work, it was reported an initial scaffold content, and chemical space diversity, and coverage” could be changed to “As part of that work, scaffold content and chemical space diversity were examined”.
    
- In the first paragraph of the “Results and discussion” section, “place of recollection” would be better stated as “place of collection”.  

In the methods section, the authors detail their procedure for “cleaning” the databases. It would be useful to others if the source code for these procedures was made available as part of the GitHub repository associated with this paper.  

 The authors used canonical SMILES to identify exact matches between compounds in different databases. While this is a reasonable approach, canonical SMILES do not standardize tautomers and may miss some duplicates. An alternate approach would be to use InChI keys, which canonicalize tautomeric forms. 

 The description of the “Consensus Diversity (CD) Plot” in the “Global diversity” section is a bit vague. An additional paragraph describing this method would be useful.  

 The density of points in Figure 3 obscures many of the points in the plot. The authors may want to consider using a plotting technique like hexagon binning which enables the plotting of large datasets without obscuring points.  For example, see these links: 

- https://datavizproject.com/data-type/hexagonal-binning/
- https://rdrr.io/cran/som.nn/man/hexbinpie.html

I’m not convinced that plotting molecular weight vs LogP is the best way to represent the diversity and overlap of datasets. An alternative might be to use a technique like t-SNE to project a set of fingerprints into a lower dimensional space: https://scikit-learn.org/stable/modules/generated/sklearn.manifold.TSNE.html.

 It might also be interesting to provide another plot which only shows the molecules from the three datasets studied that are in “drug-like” chemical space rather than the very large range shown in Figure 3. 

 In summary, this paper provides a valuable resource to researchers working in a number of different areas.  Hopefully, these suggestions will provide minor enhancements to the work.

Is the work clearly and accurately presented and does it cite the current literature?

Yes

If applicable, is the statistical analysis and its interpretation appropriate?

Yes

Are all the source data underlying the results available to ensure full reproducibility?

Yes

Is the study design appropriate and is the work technically sound?

Yes

Are the conclusions drawn adequately supported by the results?

Yes

Are sufficient details of methods and analysis provided to allow replication by others?

Yes

Reviewer Expertise:

Computational Chemistry, Cheminformatics, Drug Design

I confirm that I have read this submission and believe that I have an appropriate level of expertise to confirm that it is of an acceptable scientific standard.

# Peer review: Reviewer response for version 1

**DOI:** 10.5256/f1000research.23733.r57653

**Published:** 2019-12-13

**Authors:** Johannes Kirchmair

**Affiliations:** 1 Department of Pharmaceutical Chemistry, University of Vienna, Vienna, Austria

Sánchez-Cruz et al. report on the diversity of an extended version of BIOFACQUIM, a database of natural products extracted from organisms endemic to Mexico.

 The overall scientific approach is sound. The only technical issue that I believe requires attention is tautomerism, which appears to have been neglected. Consideration of tautomerism will unlikely change the outcomes and conclusions of the statistical analysis but for the functional group analysis it will be important to understand whether the five new functional groups from BIOFACQUIM presented in Figure 2 are indeed unique to that source. Could the authors please use InChI, MolVS or a similar notation/approach for standardizing the molecular (sub-) structures and conduct a tautomer-invariant identification of functional groups?

 Language issues are apparent throughout the manuscript and careful proofreading will be required. Some (parts of) statements are unclear, e.g.

 "it was reported an initial scaffold content"

 "the articles should have described the procedure"

 "decided to augment the number of Mexican institutions"

 "A similar procedure can be done using RDKit"

 Minor comment: Could the authors please clarify in the manuscript the sources of the natural products included in BIOFACQUIM (e.g. plants, bacteria, fungi)?

Is the work clearly and accurately presented and does it cite the current literature?

Yes

If applicable, is the statistical analysis and its interpretation appropriate?

Yes

Are all the source data underlying the results available to ensure full reproducibility?

Yes

Is the study design appropriate and is the work technically sound?

Partly

Are the conclusions drawn adequately supported by the results?

Yes

Are sufficient details of methods and analysis provided to allow replication by others?

Yes

Reviewer Expertise:

cheminformatics, natural products research

I confirm that I have read this submission and believe that I have an appropriate level of expertise to confirm that it is of an acceptable scientific standard, however I have significant reservations, as outlined above.
